# Healthcare team resilience during COVID-19: a qualitative study

**DOI:** 10.1186/s12913-024-10895-3

**Published:** 2024-04-12

**Authors:** John W. Ambrose, Ken Catchpole, Heather L. Evans, Lynne S. Nemeth, Diana M. Layne, Michelle Nichols

**Affiliations:** 1 College of Nursing, Medical University of South Carolina, Charleston, SC USA; 2 Department of Anesthesia and Perioperative Medicine, College of Medicine, Medical University of South Carolina, Charleston, SC USA; 3 Department of Surgery, College of Medicine, Medical University of South Carolina, Charleston, SC USA

**Keywords:** Resilience Engineering, Healthcare System, Healthcare Administration, COVID-19, Healthcare Team, Cohesion, Altruism, Thematic Analysis, Qualitative Research, Resilience

## Abstract

**Background:**

Resilience, in the field of Resilience Engineering, has been identified as the ability to maintain the safety and the performance of healthcare systems and is aligned with the resilience potentials of anticipation, monitoring, adaptation, and learning. In early 2020, the COVID-19 pandemic challenged the resilience of US healthcare systems due to the lack of equipment, supply interruptions, and a shortage of personnel. The purpose of this qualitative research was to describe resilience in the healthcare team during the COVID-19 pandemic with the healthcare team situated as a cognizant, singular source of knowledge and defined by its collective identity, purpose, competence, and actions, versus the resilience of an individual or an organization.

**Methods:**

We developed a descriptive model which considered the healthcare team as a unified cognizant entity within a system designed for safe patient care. This model combined elements from the Patient Systems Engineering Initiative for Patient Safety (SEIPS) and the Advanced Team Decision Making (ADTM) models. Using a qualitative descriptive design and guided by our adapted model, we conducted individual interviews with healthcare team members across the United States. Data were analyzed using thematic analysis and extracted codes were organized within the adapted model framework.

**Results:**

Five themes were identified from the interviews with acute care professionals across the US (*N* = 22): *teamwork in a pressure cooker*, consistent with working in a high stress environment; *healthcare team cohesion*, *applying past lessons to present challenges*, congruent with transferring past skills to current situations; *knowledge gaps*, and *altruistic behaviors*, aligned with sense of duty and personal responsibility to the team. Participants’ described how their ability to adapt to their environment was negatively impacted by uncertainty, inconsistent communication of information, and emotions of anxiety, fear, frustration, and stress. Cohesion with co-workers, transferability of skills, and altruistic behavior enhanced healthcare team performance.

**Conclusion:**

Working within the extreme unprecedented circumstances of COVID-19 affected the ability of the healthcare team to anticipate and adapt to the rapidly changing environment. Both team cohesion and altruistic behavior promoted resilience. Our research contributes to a growing understanding of the importance of resilience in the healthcare team. And provides a bridge between individual and organizational resilience.

**Supplementary Information:**

The online version contains supplementary material available at 10.1186/s12913-024-10895-3.

## Introduction

The COVID-19 pandemic highlighted the complexity and dynamic nature of healthcare systems. It also created a unique opportunity to look at the concept of resilience through the lens of the healthcare team versus the more common approach of situating the concept within the individual or the organization. The early phase of the pandemic was marked by challenges, such as limited access to personal protective equipment, personnel shortages, drug shortages, and increased risks of infection [1, 2]. Ensuring patient safety and proper functioning requires coordination and adaptation of the healthcare team and various processes across the health system infrastructure [3, 4]. Resilience results from adaptive coordination which enables healthcare systems to maintain routine function in the face of all conditions [5, 6].

Resilience in healthcare has been operationalized through resilience engineering, an interdisciplinary aspect of systems engineering focused on promotingpatient safety through the design, implementation, and management of healthcare systems [7–9] (e.g., how healthcare systems adapt and adjust to maneuver through the daily complexities and challenges to identify effective practices, prevent errors and maintain resilient performance) [6, 8–11]. Resilient performance in healthcare is proposed to be the net result of reaching the threshold of four resilience capabilities within the system: anticipation, the ability to expect and prepare for the unexpected; monitoring, the ability to observe threats to daily system performance; responding, the ability to adapt how the performance is enacted; and learning, the ability to learn from present and past accomplishments within the system [12]. At present, there is a paucity of research on the resilience of the healthcare team as a cohesive, singular conscious source of knowledge in a highly complex healthcare system. While the resilience of both healthcare systems [11, 13] and healthcare workers [14] has been investigated, there is a gap in knowledge specific to the resilience of the healthcare team as a unified singular consciousness. The circumstances surrounding the COVID-19 pandemic presented a unique opportunity to understand the resilience of the healthcare team in a highly complex system as a singular aware entity within the system; how it acknowledges itself, defines its purpose, and performs under extenuating circumstances. This shifts the emphasis of individual and organization resilience to the resilience in the interconnected healthcare team that extends beyond the boundary of any single person.

**Fig. 1:**
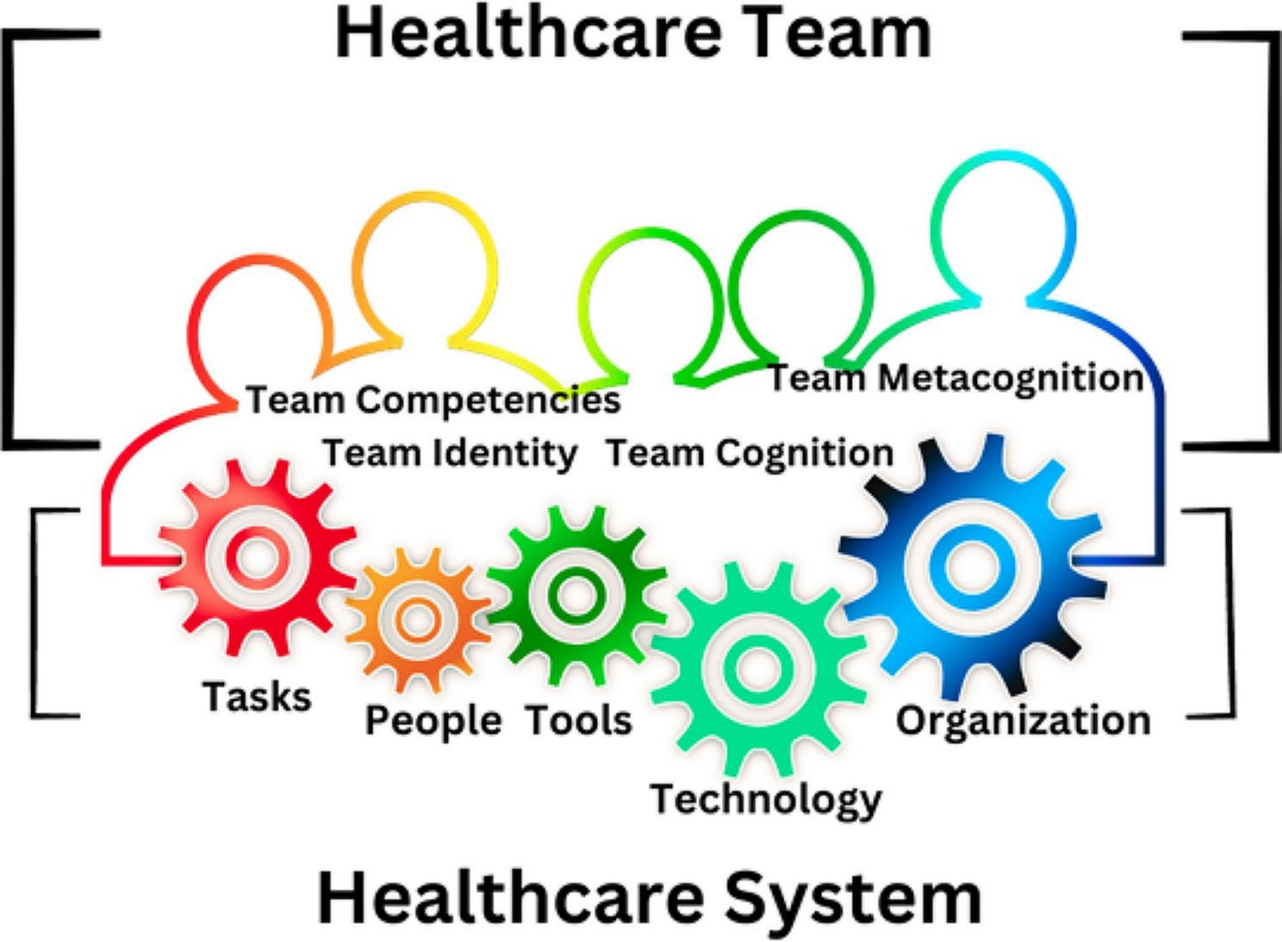
Healthcare Team as a cohesive, singular conscious source of knowledge in a highly complex system. The continuous variegated border represents the singularity and connectedness of the healthcare team within the system. The gears represent the processes, people, technology, and tasks within this highly dynamic healthcare system

The adapted model situates the healthcare team as a cohesive singlular conscious source of knowledge within an intricate and highly complex system [15]. This model was designed as a bridge between resilience found in individuals within the healthcare system and the organization to emphasize the healthcare team as an aware, unified whole. Our model [15] combines the existing Systems Engineering Initiative for Patient Safety (SEIPS) model [16] (version 1), which is based on five domains (organization, person, tasks, technologies, and tools), and environment and the Advanced Team Decision Making Model [17], which includes components for team performance [17–19]. Team performance is comprised of team identity, team cognition, team competency, and team metacognition [17–19]. Team identity describes how the team identifies their purpose to help one another [17]. Team cognition describes the state of mind of the team, their focus, and common goals [17]. Team competency describes how well the team accomplishes tasks, and team metacognition describes problem solving and responsibility [17, 19], Fig. 1.

The purpose of this qualitative research was to describe resilience in the healthcare team during the COVID-19 pandemic with the healthcare team situated as a singular conscious source of knowledge defined by its collective identity, purpose, competence, and actions. Additionally, we sought to identify factors that may facilitate or hinder the healthcare team from achieving the necessary capabilities to monitor, anticipate, adapt, and learn to meet the standard for resilient performance.

## Methodology

### Design

A qualitative descriptive design [20, 21] was employed. The interview guide was framed using the adapted model to explore various aspects of healthcare team performance (identity, purpose, competence, and cognition). These questions were pilot tested on the first 3 participants and no further changes were needed. Specifically, we aimed to investigate resilience capabilities, decision-making processes, and overall healthcare team performance.

### Sampling strategy

A purposive snowball sample was used to identify healthcare team members who worked in U.S. acute care settings between January 2020–December 2020. This sampling method was used to ensure recruitment of participants most likely to have insight into the phenomenon of resilience in the acute care setting.

#### Inclusion criteria

To explore a wide range of interprofessional experience, participants were recruited across geographic regions and professional roles through personal contacts and social media [22–25]. Eligible participants included English-speaking individuals ages 20 and older with a valid personal email address, internet access, and the ability to participate in an online video interview. Potential participants had to be employed full or part-time for any period from January 2020–December 2020 in any of the following acute healthcare environments: emergency room (ER), intensive care unit (ICU), COVID- 19 ICU, COVID-19 floor, gastroenterology inpatient unit, endoscopy suite, operating room (OR), post anesthesia recovery room (PACU), pre-operative holding area, hospital administration, or inpatient medical and/or surgical patient care unit.

#### Exclusion criteria

Healthcare team members who did not complete the pre-screening survey or failed to schedule an interview were not enrolled.

### National recruitment in the U.S

Upon approval by MUSC Institutional Review Board (IRB), registered under Pro00100917, fliers, social media posts on Twitter^TM^ (version 9.34 IOS, San Francisco, California) and Facebook^TM^ (version 390.1 IOS, Menlo Park, CA), and word of mouth were used to initiate recruitment efforts. Interested participants were sent a link to an electronic screening survey explaining the purpose of the study and verifying the respondents’ eligibility to participate. Informed consent was obtained from all subjects.

### Data collection

Data were collected via an initial screening questionnaire to determine eligibility. Data were managed using REDCap™ (version 11.2.2) electronic data capture tools hosted at MUSC. Demographic data included age, sex, race, professional role, years of experience, geographic region, patient population served, practice specialty area, and deployment status during the pandemic. Deployment refers to the reassignment of personnel from their primary clinical area to another area to meet the demands of another clinical area without regard for the participant’s clinical expertise. Qualitative data were collected through semi-structured audio video recorded interviews to understand the healthcare team in their natural environment. Recorded interviews were conducted via Microsoft® Teams (version 1.5.00.17261, Microsoft Corporation) from the PIs private office to mitigate the risk of COVID-19 transmission and promote participation across the U.S.

### Data monitoring and safety

The quality of the demographic data was monitored to ensure completeness. Potential participants who submitted incomplete responses on the questionnaire were excluded. Interviews were transcribed using software, transcriptions were reviewed and verified for accuracy, and then uploaded to MAXQDA Analytics Pro, Version 2022 (VERBI software) to facilitate data analysis. Transcripts were not returned to the participants. Qualitative codebooks, institutional review board (IRB) logs, and other study records were stored on a secure university server, with access limited to authorized study personnel. Adherence to Consolidated Criteria for Reporting Qualitative Research (COREQ) standards were maintained throughout the study and analysis [26].

### Data analysis

#### Quantitative analysis

Demographic data were analyzed using SPSS Statistics for MAC, version 28 (IBM). Both descriptive statistics for the continuous variables of age and years of experience (mean, standard deviation) and frequency tables (age, sex, race, role, geographic region, population served, deployment status) were analyzed.

#### Qualitative analysis

The Principal Investigator (PI) (JA) and senior mentor (MN) independently coded the interview transcripts. Open coding method was used to identify the categories of data [22, 27]. Both a reflexive journal and audit trail were maintained. Codes were identified through induction from participant experiences and verified through weekly consensus meetings, while theoretical deductive analysis was guided by the adapted model and the four resilience capabilities (anticipation, monitoring, responding, learning [12]. Reflexive thematic analysis (TA) [28–31] was used to analyze the coded data and generate themes. Data were collected and categorized into the codebook until no further codes were identified by the PI and research mentor [22, 27]. Participant checking was not employed.

## Results

### Demographics

The eligibility pool was established based on survey completion. Eighty-nine healthcare team members opened the online screening survey; 21 were incomplete and eliminated from the dataset, which left a pool of 68 potential eligible participants. Eligible participants (100%) were contacted by email and phone to determine their interest in completing the study interview. Twenty-two participants completed screening surveys and study interviews between May–September 2021, equating to a 32.5% enrollment rate. Participant interviews lasted between 21 and 91 min with an average of 43 min. None of the interviews were repeated. Participant demographics, including descriptive statistic and role key, are noted in Tables 1 and 2, respectively.

**Table 1:** Participant demographics

Participant demographics	*N* = 22	(n)	Percentage	Mean (SD)
Age	20–30 years	3	13.6%	46.9 (14.1)
	31–40 years	4	18.2%	
	41–50 years	5	22.7%	
	51–60 years	4	18.2%	
	> 61 + years	6	27.3%	
Years of experience	0–5 years	7	31.8%	15.8 (12.2)
	6–10 years	2	9.1%	
	11–15 years	3	13.6%	
	16–20 years	3	13.6%	
	21–25 years	1	4.5%	
	26–30 years	2	9.1%	
	> 31 years	4	18.2%	
Healthcare team role	Charge nurse	1	4.5%	
	Registered nurses	9	40.9%	
	Nurse anesthesiologists	4	18.2%	
	Leadership (manager, director, CEO)	4	18.2%	
	Physicians	3	13.5%	
	Administrative support	1	4.5%	
Racial category	American Indian or Alaskan Native	0	0	
	Asian	1	4.5%	
	Black or African American	0	0	
	Native Hawaiian or other Pacific Islander	0	0	
	White	21	95.5%	
Gender	Female	20	90.9%	
	Male	2	9.1%	
	Non-Binary	0	0	
Deployed to other Clinical Areas	Yes	11	50.0%	
	No	11	50.0%	

**Table 2:** Participant role key

Participant #	Interprofessional role
P1, DIR	Nursing director surgical services
P2, MDA	Physician anesthesiologist
P3, CRNA	Certified registered nurse anesthesiologist
P4, RN PACU	Registered nurse
P5, RN ENDO	Registered nurse gastroenterology
P6, DIR	Nursing director pediatric services
P7, CRNA	Certified registered nurse anesthesiologist
P8, ER MD	Emergency room physician
P9, ADMIN	Administrative assistant
P10,RN ENDO	Registered nurse gastroenterology
P11, CEO	Community hospital chief executive officer
P12, MDA	Physician anesthesiologist
P13, CRNA	Certified registered nurse anesthesiologist
P14, RN COVID ICU	Registered nurse COVID-19 intensive care unit
P15, CRNA	Certified registered nurse anesthesiologist
P16, RN COVID ICU	Registered nurse COVID-19 intensive care unit
P17 RN PACU	Registered nurse post anesthesia care unit
P18, RN COVID ICU	Registered nurse COVID-19 intensive care unit
P19, RN ICU	Registered nurse intensive care unit
P20,RN OR	Registered nurse operating room
P21, RN ENDO	Registered nurse gastroenterology
P22, MGR	Nurse manager preoperative services

### Themes

Five themes were identified: team*work in a pressure cooker*, *healthcare team cohesion*, *applying past lessons to present challenges*, *knowledge gaps*, and *altruistic behaviors*.

#### Teamwork in a pressure cooker

The theme *teamwork in a pressure cooker* describes the relentless pressures and emotional stressors (e.g., fear, anxiety, frustration, and stress) experienced by the healthcare team from the risks and potential threats associated with COVID-19 contamination and infection. Factors associated with these pressures included risk of COVID-19 exposure, lack of COVID-19 testing, rapid changes to policies and procedures from the standard, personnel shortages, limited physical space, and limited supplies. Exemplary quotes highlighting participant descriptions of these pressures or subthemes are noted in Table 3.

**Table 3:** Teamwork in pressure cooker: pressures faced and associated subthemes

Pressures faced	Exemplary quotes
Risk of COVID-19 exposure	“It’s actually really hard to not contaminate yourself—so I think that’s the hardest thing.” (P2, MDA)
	“We were always unprotected.” (P7, CRNA)
	“I was always very, very careful to be really good about getting my PPE right and keeping my hands clean—to do everything that I could not to catch it [COVID].” (P16, RN COVID ICU)
Lack of COVID-19 testing	“I mean, we were all exposed at one point, and I’ve never even gotten a COVID test.” (P5, RN ENDO)
	“A lot of times there’s no option to get them [nurses and doctors] tested fast enough for them to not miss their shift—I think the two biggest things that would have improved patient and staff safety and still would improve patient and staff safety are one testing capability.” (P8, ER MD)
	“What we had to be very careful— there was a shortage of test media or swabs.” (P11, CEO)
	They weren’t being tested.” (P17, RN PACU)
Rapid policy and procedure changes	“Trying to get reaccustomed to the changes that had occurred with the needs that had to be met was very difficult.” (P10, RN ENDO)
	“Things were changing fast; the CDC came out with things several times a day and we flexed and changed.” (P11, MDA)
Personnel shortages	“You need a lot more hands on deck to do it [patient care]—because there needs to be someone on the outside who’s clean and can run and get you things.” (P4, RN PACU)
	“Staff [nurses] have walked out—they can’t take it anymore.” (P2, MDA)
	“My new normal day to day—I [requested] my boss to add positions—to deal with all of the COVID stuff.” (P22, MGR)
Limited physical space	“We didn’t have enough negative air pressure rooms.” (P1, DIR)
	“You can’t socially distance in the break room, it’s not big enough.” (P6, DIR)
	“There wasn’t enough space for everybody.” (P9, ADMIN)
Limited supplies	“At first we didn’t know how much stock we had—how much was already allocated.” (P1, DIR)
	“The demand and supply [for PPE] didn’t match out ever.” (P4, RN PACU)
	“The only time I had a N95 was when I was first hired.” (P6, DIR)
	“You’re dealing with a lack of supplies—and very little support.” (P17, RN PACU)
	“We were still lacking…we were running out of PPE; we were running out of hospital beds.” (P18, RN COVID ICU)

KEY: P = Participant; DIR = Nursing Director; MDA = Physician Anesthesiologist; CRNA = Certified Registered Nurse Anesthesiologist; RN = Registered Nurse; MGR = Manager; PACU = Post Anesthesia Care Unit; ADMIN = Administrative Assistant; ENDO = Gastroenterology, CEO = Chief Executive Officer; ICU = Intensive Care Unit; OR = Operating Room

The healthcare team described an unprecedented level of stress in the workplace as the healthcare team had to adjust to rapidly changing protocols. The lack of protective equipment, shortage of providers to perform patient care and a lack of a familiar clinical routine saturated them in overwhelming pressure and emotions that stuck to them as they navigated uncharted territory. Exemplary quotes highlighting the healthcare team’s descriptions of these emotions are noted in Table 4.

**Table 4:** Teamwork in pressure cooker: emotional stressors

Emotional stressors	Exemplary quotes
Fear	“Staff were really afraid in the beginning.” (P1, DIR) “Well, in the beginning, I was really scared because, I mean, I’m in the high-risk group, you know—and I really didn’t want to be there.” (P2, MDA) “Everybody was so afraid [of COVID].” (P3, CRNA) “Everyone was very terrified, obviously, that they were going to catch it—and if they did, in fact, get it where it was going to go and how they were going to be affected or—even the long-term effects of it.” (P4, RN PACU) “The fear was the thing—you fear for your staff that you’re sending out there, fear with your taking it [COVID-19] home.” (P6, DIR) “They [staff] came in every day—if they didn’t, there wasn’t a line of people to take those jobs—people were scared.” (P11, CEO) “So, in the very beginning, I was pretty scared.” (P12, MDA) “We were all afraid of getting sick and dying.” (P15, CRNA) “Then—just like all the fear.” (P16, RN COVID ICU) “I think it [COVID] probably would have scared more people if they could have seen how bad it was, not just on the news.” (P18, RN COVID ICU) “And so, there was this fear.” (P20, RN PACU) “I’m still—on guard, because I’m afraid of the variant.” (P21, RN OR)
Anxiety	“Well, I have to say, initially it was very chaotic— the fear of the unknown.” (P5, RN ENDO) “Early on in the pandemic, there was a lot of fear and a lot of uncertainty, and there was also just constant change.” (P8, ER MD) “I guess—what I would call it, a time of uncertainty because it’s pretty much you’re going in to work every day not knowing what you’re going to see—this whole time has been about uncertainty. It’s been about you have no idea what’s coming next. You don’t know if you’re going to get it [COVID]. You don’t know if your family member is going to get it [COVID]. You don’t know hide nor hair of what you’re even doing in your job from day to day.” (P9, ADMIN) “I think we were a little unsure of what to expect because everything was so new.” (P10, RN ENDO) “It made me even more nervous because in the beginning we didn’t know.” (P12, MDA) “I just remember everything sort of like changing really quickly from shift to shift in week to week—there was so much that we didn’t know—(P16, RN COVID ICU) “It’s a pandemic—I never lived through one of those.” (P17, RN PACU)
Frustration	“It’s getting frustrating and then they change their rules that they had had established.” (P3, CRNA) “It’s just all so unknown that it’s frustrating that anyone can even think they can make a decision on what do we do about this.” (P4, RN PACU) “Staff felt a little frustrated that other people weren’t stepping up and volunteering [to be deployed] but they were.” (P6, DIR) “Never really knew what [COVID non-COVID patient assignment] you were going to get —just luck of the draw.” (P19, RN ICU) “They would have no idea what shifts we were working—it was very, very discombobulated.” (P21, RN OR) “You didn’t sign up for it, you just got handed this new role—It’s never been done before—there’s no there’s no guide on your path.” (P22, MGR)
Stress	“I just think they were so stressed on trying to keep people alive that they weren’t able to actually toilet people, then brush their hair and make sure that their activities of daily living were being met.” (P5, RN ENDO) “It was stressful—the not knowing—the level of stress that’s embedded in all of this, it complicates things (P7, CRNA ) “They were as stressed as everybody else was—they were trying their hardest to protect patients and staff.” (P11, CEO) “I feel that people knew that everyone was under a significant amount of pressure and stress.” (P15, CRNA)

KEY: P = Participant; DIR = Nursing Director; MDA = Physician Anesthesiologist; CRNA = Certified Registered Nurse Anesthesiologist; RN = Registered Nurse; MGR = Manager; PACU = Post Anesthesia Care Unit; ADMIN = Administrative Assistant; ENDO = Gastroenterology, CEO = Chief Executive Officer; ICU = Intensive Care Unit; OR = Operating Room

“It was…uncharted territory for me.” (P1, DIR)

“You were stuck in a situation you never— you didn’t know when it was going to end.” (P4, RN PACU)

“They have not enough staff—they can’t do it—they—I don’t know what we’re going to do.” (P6, DIR).

“When we deployed—trying to get re-accustomed to the changes—with the needs that had to be met was very difficult.” (P10, RN ENDO)

“I wasn’t about to sign up for extra time working in under those stressful conditions.” (P17, RN PACU)

The fear of the unknown, combined with the constant need to adapt to rapidly changing circumstances, led to widespread stress, frustration, anxiety, and exhaustion within the healthcare team. This theme was characterized by the constant pressure both inside and outside of work experienced by the healthcare team.

“Driving to the hospital, crying, driving back from the hospital, crying, still doesn’t sum it up— surrounded by people who were just dying. And what could you do?” (P6, DIR)

“It was constant. It was terrible. I couldn’t sleep at night. I’d wake up worried.” (P8, ER MD)

“It was kind of like just keep sending the Calvary forward—and when one drops, you just walk over them.” (P17, RN PACU)

“It was always there—COVID here, COVID there—you never could just completely get away from it. It was basically the center of everybody’s conversation everywhere you went or if you were on the phone with somebody.” (P18, RN COVID ICU)

“I was having to call my parents before I’d leave my apartment to go into work— to vent to them and cry— to let out my frustration and my anxiety—and have them essentially convince me to go into work.” (P19, RN ICU).

“Working so much— COVID was all that was on my brain—and it was a lot of pressure.” (P22, MGR)

Working during COVID-19 challenged the resilience of the healthcare team in the face of constant fear and uncertainty. The pressure to maintain team performance, while dealing with constant fear associated with the pandemic effected the healthcare team’s resilience.

“I have to tell you that after being in hospital—I don’t feel resilient right now— doing all the things I’ve done—I just want to be out of the hospital— [crying] I can tell you that it will stay with me the rest of my life— It will always stay with me.” (P6, DIR)

“I feel like my team has used up all of their resilience. I don’t think there’s much left.” (P8, ER MD)

However, one team member stood out as an exception. They reported the pressures from the environment helped them to make decisions. This demonstrates that environmental pressures affect members of the healthcare team differently. They reported that the pressure and intensity of the situation sharpened their focus and allowed them to make choices more quickly and effectively.

“I make better decisions when I’m under pressure.” (P22, MGR)

#### Healthcare team cohesion

The theme *healthcare team cohesion* describes the unique experience of working together during the pandemic that created a means among the healthcare team to form close relationships and unite. This bond was characterized by the emergence of strong interpersonal connections among healthcare professionals during the COVID-19 pandemic. These connections shaped healthcare team relationships and were a factor in the collaborative decision-making processes within healthcare team for their day-to day functions. This cohesive bonding was fueled by the stress and uncertainty of the situation, which brought the healthcare team together illustrated by their solidarity, camaraderie, trust, and empowerment.

“All those decisions, important decisions were made together.” (P7, CRNA)

“Everyone felt like they were they were, you know, in a in a battle zone and on the same side—and so that kind of brought people together.” (P8, ER MD)

“I think our team worked as one.” (P11, CEO)

Solidarity described the sense of unity evident among the members of the healthcare team. This was characterized by connectedness and a sense of reliance on one another that promoted teamwork and resilience within the team from support both given and received. The sub-theme camaraderie described the close personal connection and support between the healthcare team that went beyond normal social interactions prior to the pandemic. These connections were filled with trust and respect for other healthcare team members.

“I think we were all trying to do the best we could do and help each other do the best they could do—I think early on just camaraderie helped a lot within the department and, you know, just relying on each other for support.” (P8, ER MD)

“We knew that we can depend on each other and we all had different skill sets— I think that that was very important—that made us feel secure— rather than going alone.” (P10, RN ENDO)

“We [The ICU Nurses] developed a sense of camaraderie that I mean, it’s nothing I’ve ever felt before, like we had to trust each other with our licenses, with our own health—my resiliency came from my coworkers.” (P14, CHG RN)

“One of the things that I think the pandemic did in a positive—was—I believe that the teams that I worked for really started to solidify. We leaned on each other. I felt more of a team environment than I had had pre-pandemic—I felt that people were a bit better together. We all needed each other, and we all leaned on each other, and we gave each other support—more so than before COVID- 19.” (P15, CRNA)

”The nurses on the unit were always there for me—they became my friends— my family.” (P19, RN ICU)

The sub theme of empowerment referred to the ability of the healthcare team to confidently make decisions and assume responsibility for their actions within the healthcare setting. This process involved a sense of authority and the ability to exercise agency in decision-making together to respond and adapt to the demands the healthcare team experienced. The combination of solidarity, camaraderie, trust, and empowerment resulted in a strong sense of cohesion within the healthcare team which led to improved relationships and enhanced resilience in their performance.

“I felt that I felt that the team—we all needed each other and we all leaned on each other and we gave each other support—more so than before COVID.” (P15, CRNA)

“How do you want to handle this? What’s the plan?—and we collaborated in the true sense of collaboration.” (P15, CRNA)

“We just knew that we could count on each other—we knew that we could count on each other at any time if we had questions, because we all worked so closely together during this. We really became a really tight knit group, and it was great.” (P22, MGR)

The benefits of the cohesion found in the healthcare team were significant and apparent during the COVID-19 pandemic. The strengthened relationships and increased resilience allowed for improved communication and collaboration, leading to better patient care and outcomes. Despite these advantages, it was noted by one participant that the relationships developed were not sustained beyond the peak of the pandemic.

“Now that COVID is kind of at bay in our area, it’s kind of gone back to the same way it was— it has not stuck.” (P15, CRNA)

#### Applying past lessons to present challenges

The theme *applying past lessons to present challenges* describes how the knowledge and understanding gained from prior participant experiences was used to adapt to the novel clinical and infrastructural challenges faced during the pandemic. Past experiences facilitated the healthcare team to strategize ways to meet the demands of the healthcare system during this time.

Participants described two strategies the healthcare team used to improve the system’s ability to adapt and function effectively: changing roles and deploying personnel. The process of changing roles involved assigning new responsibilities to individuals based on priority-based initiatives, while deployment involved transferring clinical staff from areas with lower patient care needs to those with higher needs to optimize their utilization. Eleven participants (50%) were affected by these strategies. Of these, 73% were assigned to clinical areas for direct patient care, while the remaining 27% underwent a role change to support the operational needs of the system. The participants’ preexisting work relationships, specialized clinical expertise, and leadership abilities helped them adapt to their new clinical and non-clinical roles, which in turn enhanced the resilience of the healthcare team.

“We wanted to make sure that we were putting people into the right area where their skill set could be used the best.” (P1, DIR)

“I’m known for moving people forward—I’m also well known for speaking up when I don’t think it is right and there was a lot of stuff that I didn’t think was right— and not only speaking up, I’m also going to come with the solution.” (P6, DIR)

Participants indicated the lessons learned from prior experience positively impacted team performance and improved patient care outcomes. There were two significant examples in the data: the perspective of a nurse who was redeployed to work in an obstetrics unit (P5, ENDO RN) and the perspective of a nursing director (P6, DIR) whose role was changed to develop a program to ensure adequate staffing.

“Because we [the team of interprofessionals] were all very familiar with what we had to do at the task, at handit [the experience of the provision of care] was very fluid—I think it’s because of our years of experience and working with each other for so long that it just worked out very well ”. (P5, ENDO RN)

“Staff believed in me when I said I would do something— I could galvanize people because of my reputation of caring for staff, so I was chosen specifically because of my ability to move people forward in spite of things.” (P6, DIR)

Participants identified being assigned to unfamiliar clinical areas or working with unfamiliar patient populations as a barrier that hindered their ability to adapt to clinical situations. The lack of clinical competence among some personnel led to an increase in workload for other healthcare team members, who had to provide additional instruction and guidance on how to complete the task. Decision-makers who deployed nursing staff to a clinical area with higher staffing needs may have believed that the individual nurse had specific clinical skills that would be helpful in that area, and this was not the case.

“She [the patient] felt like it was that he [the new nurse]—really didn’t know what he was doing—not only were we kind of reintroduced to that role of caring for patients where we haven’t been recently, but we’re also in a teaching mode, too, for the new nurses—we had to prioritize how sick the patients were, from basic vital signs to wound dressings to respiratory, and help those new nurses know which to attend to first.” (P10, RN ENDO)

“Nurses weren’t really put in a place with enough support and enough resources to be able to do a job, and to do a job that maybe they haven’t done for a few years.” (P10, RN ENDO)

The participants indicated that clinical competencies of a healthcare provider in one patient population may not necessarily be applicable to another patient group. For instance, a neonatal intensive care unit (NICU) nurse who has experience in managing Extra Corporeal Membranous Oxygen (ECMO) in newborns may not have the necessary skills to care for adult ECMO patients in an adult COVID-19 intensive care unit.

“The ECMO nurse was a NICU nurse, so she really could not help me.” (P14, CHG RN)

#### Knowledge gaps

The theme *knowledge gaps* refers to the disparity between the existing knowledge of the healthcare team and the knowledge required for the team to effectively respond and adapt to the needs of the healthcare system. The lack of COVID-19 specific knowledge led to gaps in the healthcare team’s understanding, while the lack of communication made it difficult for necessary information to be effectively conveyed and received (e.g., medical logistics, human resources, and other operational policies and procedures). This knowledge gap created a barrier to healthcare team resilience as their capacities to surveil, anticipate, and respond were diminished from the lack of knowledge.

“That [information] is pretty fundamental to how you [the healthcare team] function.” (P17, RN PACU)

“I don’t think any amount of preparation could have actually prepared us for how bad COVID was—but we were very, very, very unprepared.” (P18, RN COVID ICU)

“It was confusing, it was disruptive to the patients that we had there. They sensed that. And that’s— OK—screw with me, screw with my colleagues, but don’t screw with the patient.” (P21, RN ENDO)

All the participants in leadership roles during the COVID-19 pandemic emphasized the importance of having a thorough understanding of the information and effectively communicating it to the frontline healthcare team members most involved in providing patient care.

“There’s nothing worse than having to learn something in the moment and not being prepared for it.” (P1, DIR)

“That made us communicate in multiple ways throughout a day because we all know people learn and adapt it could be in print. It could be in person; it could be a video. We tried to have multiple ways of getting messages out and knowing we needed to repeat messages because this was so unknown, and people were so stressed.” (P11, CEO)

One team member (P13, CRNA), highlighted areas where there were gaps in knowledge in greater detail.

“It was as if the unit was being run by all these sort of substitute teachers that were called in at the last minute. Nobody knew where stuff was—nobody knew what the protocol was—the communication was terrible.” (P13, CRNA)

The cumulative effect from the knowledge gaps contributed to the lack of a practical working knowledge for the healthcare team and affected the healthcare team’s ability to anticipate what needed to be done and adapt their performance to accomplish it. Despite knowledge gaps, healthcare team members reported their capability to learn was facilitated by incremental gains in practical knowledge through their experience over time.

“—people got to be experts at protecting patients and keeping themselves safe.” (P8, ER MD)

“I think it kind of was like on the job training at that point, I felt like we were all just trying to survive—learning was like—you went out —then you came back, and you would share how things went.” (P15, CRNA)

“You tried to educate yourself so you could be safe.” (P17, RN PACU)

The participant responses received from the leadership (CNO, Directors, and Manager) and front-line personnel (administrative staff, nurses, and physicians) regarding the importance of communication highlighted a difference in perspective. Leadership exhibited a strong commitment toward effective communication and made efforts to ensure all healthcare team members were well informed. On the other hand, the frontline participants indicated instances where communication strategies were not perceived as effective.

“I wasn’t contacted by a manager from the unit or anything to be able to reassure, reassure me that things were being followed through and it should be okay, so that was tough.” (P10, RN ENDO)

“It really seemed like there was no communication between—like staffing and the floor—we would get up to the floor and they would say, who are you? What are you doing here? What are we supposed to do with you?” (P20, RN OR)

#### Altruistic behaviors

The theme *altruistic behaviors*, encompasses the participants’ perception of their obligation and accountability to their patients and healthcare team, and their steadfastness in supporting the healthcare team even if it meant facing personal or professional repercussions. This readiness to aid the healthcare team and accept consequences showcased their altruism and commitment to the healthcare team. The team’s dedication to both their patients and each other was a primary focus driven by a strong sense of responsibility and obligation.

“I want to be able to look myself in the mirror and feel like I did the right thing—.” (P6, DIR)

“My resiliency came from my coworkers. I wanted to come back to work to help them.” (P14, RN COVID ICU)

“People really looked out for each other—and people were really kind and compassionate to each other—we all were in this together.” (P15, CRNA)

“I’m grateful for the experience that I had and all of the different patients that I was able to help in my time there definitely solidified that being a nurse is what I needed to do—and why I chose the profession is exactly what I should have been doing.” (P19, RN ICU)

“You just have to go with what seems right—.” (P22, MGR)

A defining characteristic of this theme was a willingness to endure consequences for the benefit of the healthcare team. These consequences varied from contracting the virus, facing criticism from the healthcare team, to foregoing financial incentives, and even job loss.

“I felt like I was punished for speaking up and I was punished for doing the right thing for patients.” (P6, DIR)

“I mean, I literally broke the law so many times. Do you know how many times I started pressors [vasoactive drugs to increase blood pressure] on patients that I had no orders for [because a physician would not enter the ICU]?” (P14, CHG RN)

## Discussion

We identified five key themes based on the coded data; namely *teamwork in a pressure cooker*, *healthcare team cohesion*, *applying past lessons to present challenges*, *knowledge gaps*, and *altruistic behaviors*. The researchers propose that stressors arising from the COVID-19 pandemic had an impact on the healthcare team’s resilience. In addition, strong healthcare team cohesion, selfless behaviors among the healthcare team, shared knowledge, and job competence within the healthcare team, enhanced resilient performance.

The healthcare team experienced significant stress and uncertainty, due to the COVID-19 pandemic. This is consistent with previous research that has shown that the unprecedented nature of the pandemic led to challenging working conditions, limited resources, lack of information, and concerns about infecting loved ones [32–44]. The collective global impact of COVID-19 on healthcare systems is likely a contributing factor to these stressors [45–48].

Our study, along with those conducted by Anjara et al. (2021)[49] and Kaye-Kauderer et al. (2022) [50], found that solidarity and camaraderie among healthcare professionals improve resilience. Specifically, Anjara et al. observed increased collaboration among the healthcare professionals they studied in Ireland during the COVID-19 pandemic, while Kaye-Kauderer et al. identified team camaraderie among their sample of front-line healthcare workers from New York. Kinsella et al. (2023) [51] reported that COVID − 19 offered frontline workers in the UK the opportunity to work together toward a common goal. Potential explanations for these findings align with the concepts of social capital proposed by Coleman [52] and social identification with other as proposed by Drury [54]. Coleman suggests an individual’s skills and capabilities are enhanced through their interdependent relationships with others [52]. Drury found in communities affected by disasters, mutual aid and support emerged from a shared social identity, which serves to strengthen the community [53]. Brooks et al. (2021) [54] conducted a study with healthcare, police, and commercial sectors in England. They found it was important for these individuals to receive support from and provide support to their colleagues to mitigate the psychological impact of disaster exposure [54]. In addition, like our findings, Aufegger and colleague’s 2019 systematic review [55] found that social support in acute care healthcare teams creates a supportive atmosphere where team members help each other communicate problems, fulfill needs, and deal with stress.

Our results are consistent with those of Liu et al. (2020) [32] and Banerjee et al. (2021) [44] who each found that healthcare professionals frequently feel a sense of personal responsibility to overcome challenges. One potential explanation for this may be the influence of collectivism in their cultures. Similarly, our study suggests the sense of camaraderie among healthcare professionals may also contribute to a sense of responsibility and increased altruistic behavior. However, other studies have highlighted different perspectives on healthcare professionals’ sense of responsibility and duty. Godkin and Markwell’s (2003) [56] revealed that healthcare professionals’ sense of responsibility during the Severe Acute Respiratory Syndrome (SARS) outbreak was dependent on the protective measures and support offered by the healthcare system where most SARS infected patients were hospitalized. More recently, Gray et al. (2021) reported that nurses’ sense of responsibility stems from their ethical obligations, regardless of potential personal or familial risks [57].

The altruistic behaviors described by our participants helped maintain the performance of the healthcare team. It is too soon to see the long-term impact from working in this high-pressure environment; however, past research by Liu et al. (2012) [58] and Wu (2009) [59] demonstrated that “altruistic-risk acceptance” during the SARS outbreak was shown to decrease depressive symptoms among hospital employees in China.

Our research on resilience has important implications for healthcare organizations and professionals. In order to ready themselves for forthcoming events, healthcare systems must emphasize the significance of shared knowledge and its influence on the healthcare team’s ability to foresee and monitor effectively. This knowledge can help the healthcare organization function as a unified entity, rather than as individuals in separate roles or clusters within the organization to improve healthcare team preparedness. Establishing a cohesive, clinically competent healthcare team benefits the organization and the patients served. Measures to enhance social support, improve communication and ensure clinical competence maintain healthcare team resilience.

There are several limitations to consider when interpreting the results of this study. First, the sample was obtained using purposive snowball sampling, which may have introduced sampling bias and may not accurately represent the larger population of healthcare team members who worked during the COVID-19, as 95% of the sample were white. Second, our study did not have equal representation of all interprofessional team members. It is possible that a more heterogenous sample regarding role, race and gender may have introduced additional codes. Additionally, the PI (JA) worked as a Certified Registered Nurse Anesthesiologist (CRNA) in acute care during the pandemic and personal experience may have introduced confirmation bias. Also, the focus of our research was to fill a gap in the existing knowledge of what is known about healthcare team resilience in pandemic disasters, and help to answer if and how it intersects with individual and organizational resilience. It is possible this novel conceptualization of healthcare team as a cohesive singular conscious source of knowledge did not adequately address this.

Steps to ensure rigor and mitigate any potential shortcomings of qualitative data analysis were the maintenance of a reflexive journal, a willingness of the PI to let go of unsupported ideas and constant verification of codes and themes with the research mentor (MN) for coherence and consistency within the coded data, selected methodology and research questions.

## Conclusion

Overall, the extracted themes of *teamwork in a pressure cooker; healthcare team cohesion; applying past lessons to present challenges; knowledge gaps; and altruistic behaviors* illustrate comparable experiences within the healthcare team. As healthcare professionals and organizations continue to navigate the challenges of the COVID-19 pandemic and other crises, our findings provide valuable insights into how team cohesion, along with altruistic behaviors, may enhance resilience capabilities to create and maintain a unified resilient healthcare team.

### Electronic supplementary material

Below is the link to the electronic supplementary material.

Supplementary Material 1

## Data Availability

The data for this study are confidential as required by the IRB approval. To protect the anonymity of the participants, the data are not publicly available. Additional information about the research method, Interview questions, informant data, and the study in general can be requested from the corresponding author, J.A.
