# Supplementary material for: Healthcare team resilience during COVID-19: a qualitative study
Source: BMC Health Serv Res. 2024 Apr 12;24:459. doi: 10.1186/s12913-024-10895-3 (PMC11010334; doi:10.1186/s12913-024-10895-3)
Supplement: Supplementary file 1 — Supplementary Material 1 [file 12913_2024_10895_MOESM1_ESM.docx]

Consolidated criteria for reporting qualitative studies (COREQ): 32-item checklist

Please indicate in which section each item has been reported in your manuscript. If you do not feel an item applies to your manuscript, please enter N/A.

For further information about the COREQ guidelines, please see Tong *et al.*, 2017: <https://doi.org/10.1093/intqhc/mzm042>

| **No.** | **Item** | **Description** | **Section #** |
| --- | --- | --- | --- |
| **Domain 1: Research team and reflexivity** | | | |
| Personal characteristics | | | |
| *1.* | Interviewer/facilitator | Which author/s conducted the interview or  focus group? | Page 9 line 170-1 |
| *2.* | Credentials | What were the researcher's credentials? *E.g.*  *PhD, MD* | Page 9 |
| *3.* | Occupation | What was their occupation at the time of the  study? | Page 9,31 |
| *4.* | Gender | Was the researcher male or female? | Page 9 |
| *5.* | Experience and  training | What experience or training did the researcher  have? | Page 9 |
| Relationship with participants | | | |
| *6.* | Relationship  established | Was a relationship established prior to study  commencement? | Page 7 |
| *7.* | Participant knowledge of the interviewer | What did the participants know about the researcher? *E.g. Personal goals, reasons for*  *doing the research* | Page 7 |
| *8.* | Interviewer characteristics | What characteristics were reported about the interviewer/facilitator? *E.g. Bias, assumptions,*  *reasons and interests in the research topic* | Page 31 |
| **Domain 2: Study design** | | | |
| Theoretical framework | | | |
| *9.* | Methodological orientation and theory | What methodological orientation was stated to underpin the study? *E.g. grounded theory, discourse analysis, ethnography,*  *phenomenology, content analysis* | Page 6 |
| Participant selection | | | |
| *10.* | Sampling | How were participants selected? *E.g. purposive,*  *convenience, consecutive, snowball* | Page 6 |
| *11.* | Method of approach | How were participants approached? *E.g. face-*  *to-face, telephone, mail, email* | Page 7 |
| *12.* | Sample size | How many participants were in the study? | Page 9 |
| *13.* | Non-participation | How many people refused to participate or  dropped out? What were the reasons for this? | Page 9 |
| Setting | | | |
| *14.* | Setting of data  collection | Where was the data collected? *E.g. home, clinic,*  *workplace* | Page 8 |
| *15.* | Presence of non-  participants | Was anyone else present besides the  participants and researchers? | Page 8 |

| *16.* | Description of sample | What are the important characteristics of the  sample? *E.g. demographic data, date* | Page 10 |
| --- | --- | --- | --- |
| Data collection | | | |
| *17.* | Interview guide | Were questions, prompts, guides provided by  the authors? Was it pilot tested? | Page 6 |
| *18.* | Repeat interviews | Were repeat interviews carried out? If yes, how  many? | Page 10 |
| *19.* | Audio/visual recording | Did the research use audio or visual recording  to collect the data? | Page 8 |
| *20.* | Field notes | Were field notes made during and/or after the  interview or focus group? | Page 9 |
| *21.* | Duration | What was the duration of the interviews or  focus group? | Page 10 187-189 |
| *22.* | Data saturation | Was data saturation discussed? | Page 9 177-179 |
| *23.* | Transcripts returned | Were transcripts returned to participants for  comment and/or correction? | Page 8 |
| **Domain 3: analysis and findings** | | | |
| Data analysis | | | |
| *24.* | Number of data  coders | How many data coders coded the data? | Page 9 |
| *25.* | Description of the  coding tree | Did authors provide a description of the coding  tree? | Page 9 |
| *26.* | Derivation of themes | Were themes identified in advance or derived  from the data? | Page 9 |
| *27.* | Software | What software, if applicable, was used to  manage the data? | Page 8 Line157 |
| *28.* | Participant checking | Did participants provide feedback on the  findings? | Page 9 line |
| Reporting | | | |
| *29.* | Quotations presented | Were participant quotations presented to illustrate the themes / findings? Was each  quotation identified? *E.g. Participant number* | Pages 12-28 |
| *30.* | Data and findings  consistent | Was there consistency between the data  presented and the findings? | Pages 31-32 |
| *31.* | Clarity of major  themes | Were major themes clearly presented in the  findings? | Pages 12-38 |
| *32.* | Clarity of minor  themes | Is there a description of diverse cases or  discussion of minor themes? | Page 15,19 |

When submitting your manuscript via the online submission form, please upload the completed checklist as a Figure/supplementary file.

If you would like this checklist to be included alongside your article, we ask that you upload the completed checklist to an online repository and include the guideline type, name of the repository, DOI and license in the *Data availability* section of your manuscript.

Developed from: Allison Tong, Peter Sainsbury, Jonathan Craig, Consolidated criteria for reporting qualitative research (COREQ): a 32-item checklist for interviews and focus groups, International Journal for Quality in Health Care, Volume 19, Issue 6, December 2007, Pages 349–357, <https://doi.org/10.1093/intqhc/mzm042>
